# Vaginal Ulcers Secondary to Stage Iv Uterine Prolapse Treated With L‐PRF

**DOI:** 10.1002/ccr3.72098

**Published:** 2026-02-24

**Authors:** Alberta Greco Lucchina, Enrico Rescigno, Canata Alessandra, Gianluca Nicolai, Antonio Scarano

**Affiliations:** ^1^ Research Laboratory in Regenerative Medicine and Tissue Engineering, Saint Camillus International University of Health Sciences Rome Italy; ^2^ Lavagna Hospital (Ge) ASL 4 Liguria Italy; ^3^ General practitioner Lavagna (Ge) ASL 4 Liguria Italy; ^4^ Department, Maxillo‐Facial Surgery Università di Tor Vergata Rome Italy; ^5^ Department of Innovative Technologies in Medicine & Dentistry University of Chieti‐Pescara Cheti Italy

**Keywords:** endocrine dysfunction, pelvic prolapse, platelet‐rich plasma, reproductive, uterine prolapse

## Abstract

Pelvic organs prolapse (POP) is usually used to describe the pelvic organs' descent into or through the vagina. The related common complications are bleeding, extrusion, vaginal discharge, pain, and constipation; only older women and those with comorbidities seem to be most prone to persist with their use. Surgery is preferred, instead, by younger active women with advanced prolapse symptoms. The uterus lowers and slides into the vagina. Treatment varies by stage and severity. The purpose of this article was to evaluate the efficacy of LPRP in the treatment ofuterine ulcers in an old 93 years' obese patient with UP stage IV non‐surgically treatable. Obese 93‐year‐old woman with medical history of left hip replacement, bilateral knee replacement COPD with chronic pulmonary heart disease, renal failure, chronic heart failure, one hospitalization for acute heart failure, suffering from umbilical hernia, hypertension, hyperuricemia, polyhydric arthrosis, has had a complete uterine prolapse (grade IV) for 5 years. The patient has multiple vaginal ulcers as a consequence of urinary and fecal incontinence fluid in continuous contact with the occasionally inflamed everted vaginal mucosa. The ulcer area was infiltrated with L‐PRF membranes and covered with PRF, which were sutured and protected with an inverted glove. The results of the present case report show the positive effect of L‐PRF on the uterine ulcers that is a promising treatment for vaginal ulcers in case of prolapse. In conclusion, L‐PRF has shown significant potential as an effective treatment for chronic non‐healing uterine ulcers, offering advantages over conventional dressings in terms of efficacy, safety, and cost‐effectiveness.

## Key Clinical Message

The purpose of this article was to evaluate the efficacy of LPRP in the treatment of vaginal ulcers in an old 93 years' obese patient with UP stage IV non‐surgically treatable. In conclusion, L‐PRF has shown significant potential as an effective treatment for chronic non‐healing uterine ulcers, offering advantages over conventional dressings in terms of efficacy, safety, and cost‐effectiveness.

## 1 Introduction

The term, pelvic organ prolapse (POP) is usually used to describe the pelvic organs' descent into or through the vagina. Uterine prolapse (UP) is one of the multiple conditions that are characterized under the generic name POP.

The herniation of the uterus through the vaginal canal [1] is a condition derived from the weakness in the supporting structures of the pelvic floor.

Delancey proposed a subdivision of the female pelvic structure in three levels [2]. Level 1 represents the cardinal‐uterosacral ligament complex, which provides attachment of the uterus and vaginal vault to the bony sacrum. Level 2 and 3 consist of fascia, the urogenital diaphragm and the perineal body supporting the middle and lower part of the vagina.

In physiological conditions, the uterus and the vagina are suspended through the cardinal‐uterosacral ligament complex from the sacrum and lateral pelvic sidewalls.

The disruption and loosening of such fundamental structure, due to qualitative and quantitative changes in connective tissues [3], facilitate the uterus descent into the vaginal vault, eliciting the condition.

UP is not a life‐threatening condition, but it can be associated with inability to work, difficulties in walking or standing up, urinating or defecating, painful intercourse, or social stigma [4]. Among the wide range of symptoms usually reported [5], only vaginal bulge seems to be the most specific for UP, and other conditions, such as urethral diverticulum, rectal prolapse, or vaginal cysts, should be considered in the differential diagnosis [6].

The worsening of the symptoms seems also to be directly correlated to the severity of the prolapse [7].

In developing countries UP represents the most reported health issue contributing to morbidity and mortality in women of reproductive age [4]. The etiology is complex and multi‐factorial. Aging, menopause, delivery history, smoking, chronic pulmonary disease are hypothesized by Olsen et al. [8] as being potential risk factors [9]. In addition, higher ORs were reported in overweight and obese women [8, 9, 10].

Given that the US, Europe, and Australia are facing increasing obesity rates and an aging population, the prevalence and severity of UP is expected to increase over the coming years [11]. Connective tissue disorders, such as Marfan or Ehlers‐Danlos syndrome, are also thought to have an active role in UP pathogenesis [12]. Demographic epidemiology studies also support the hypothesis that ethnic and racial variations could have a relevant impact on the manifestation of the condition [13].

The epidemiology of UP is difficult to estimate since many studies do not distinguish between prolapse of all pelvic organs and uterus prolapse.

In a cross sectional study of 1961 women, 9.7% between ages 20%–39% and 49.7% > 80 years old complained at least 1 pelvic floor disorder [14]. In the WHI trial, 14% of women enrolled showed some degree of UP [15]. A UK study, following more than 17 k women aged 25–39, reported an annual incidence of surgery for prolapse of 2,04 per 1000 patients [16]. Prolapse is a typical issue caused by the uterus's supporting structures gradually deteriorating. An estimated 30% of women over fifty are thought to suffer from uterine prolapse [17]. Neonatal birthweight and duration of labour are independent factors that increase the risk of uterine prolapse. An important trigger is increased intra‐abdominal pressure such as obesity, bronchiectasis, chronic bronchitis, asthma, and constipation and may move the integrity of the pelvic floor. In 1996 the standardized system POP‐Q for evaluating POP was introduced to evaluate the degree of pelvic floor dysfunctions [18]. To overcome the specificity and the perceived difficulty in using this system, a simplified version (S‐POP‐Q) was also proposed [19].

Even if POP‐Q represents the most used system, other previous classification tools such as the Baden‐Walker halfway system have maintained a remarkable popularity among clinicians [20].

Conservative treatments, such as pelvic floor physical therapy (PPFT) and vaginal pessaries, showed to be effective solutions for the subjective improvement in UP symptoms [21] and progression [22], especially in early stages. Pessary should be considered a successful first line treatment. However, owing to the related common complications (bleeding, extrusion, vaginal discharge, pain, and constipation) [23] only older women and those with comorbidities seem to be most prone to persist with their use. Surgery is preferred, instead, by younger active women with advanced prolapse symptoms [24]. The uterus lowers and slides into the vagina. The stages of uterine prolapse are often classified into stages based on severity in four stages: uterine prolapse is classified into four stages based on the extent of descent. Stage I involves the most distal portion of the prolapse being more than 1 cm above the hymen. Stage II is when the prolapse is between 1 cm above and 1 cm below the hymen. Stage III is characterized by the prolapse extending more than 1 cm below the hymen but not completely protruding. Stage IV, or complete prolapse, involves total vaginal vault eversion [25]. Treatment varies by stage and severity. Conservative management includes pelvic floor exercises and pessaries, while surgical options range from uterine‐sparing procedures to hysterectomy. Minimally invasive techniques, such as transposition of cardinal ligaments, are also employed for stages II–III to preserve fertility and reduce operative time [26].

Different surgical techniques have been proposed for the treatment of such Vaginal meshes and injectable PRP, which could predominantly enforce the ligaments. The purpose of this article was to evaluate the efficacy of LPRP in the treatment vaginal ulcers in an old 93 years' obese patient with of UP stage IV non‐surgically treatable.

## 2 Case History/Examination

**FIGURE 1:**
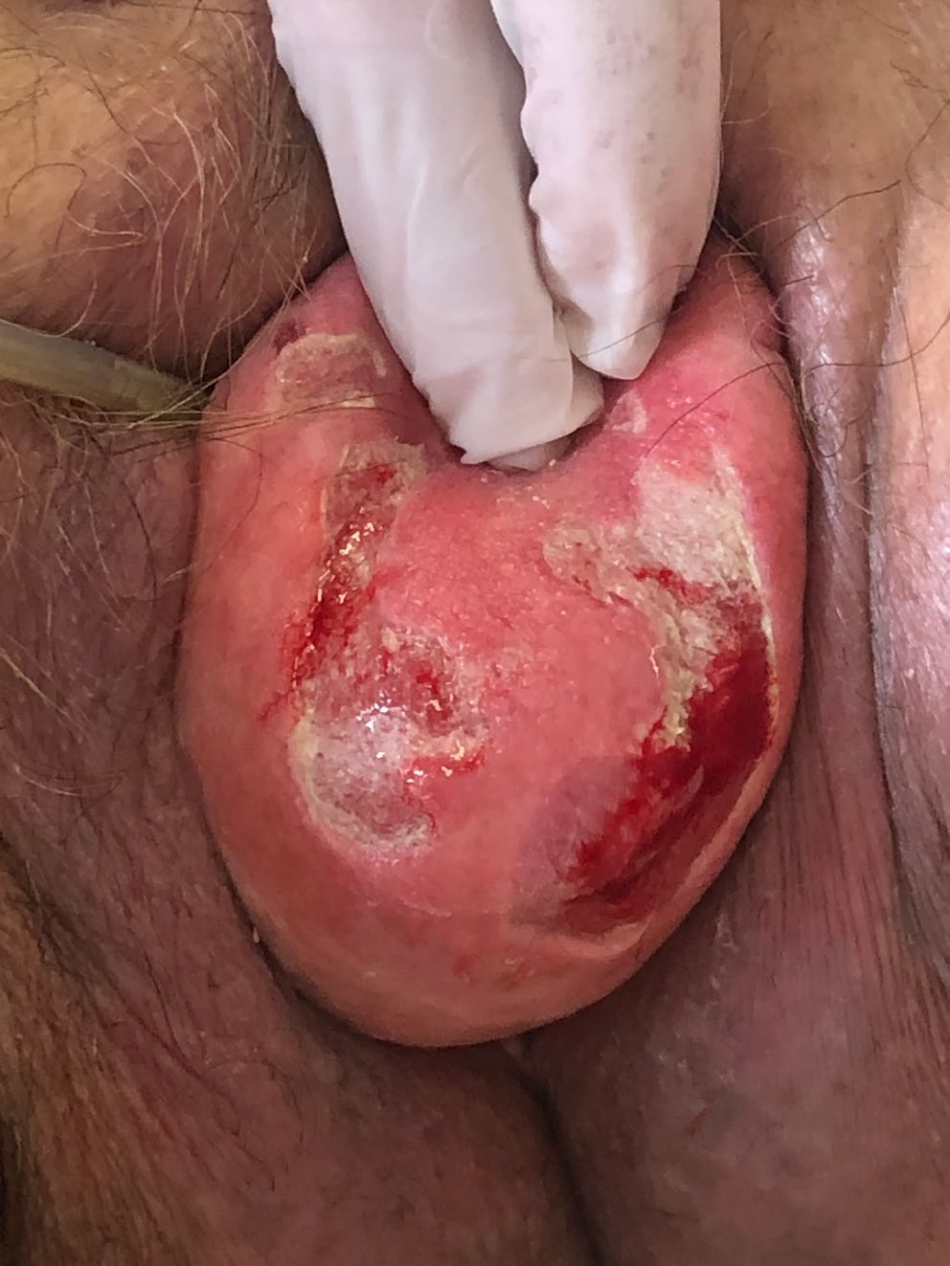
Complete uterine prolapse grade IV with two ulcers.

Obese 93‐year‐old woman with a medical history of left hip replacement, bilateral knee replacement, COPD with chronic pulmonary heart disease, primary renal failure, chronic heart failure, one hospitalization for acute heart failure, suffering from umbilical hernia, hypertension, hyperuricemia, polyhydric arthrosis, has had a complete uterine prolapse (grade IV) for 5 years (Figure 1). On 2 February 2021, she was hospitalized in the pathology clinic due to a urinary tract infection and severe deterioration of renal function. The patient has multiple vaginal ulcers as a consequence of urinary and fecal incontinence fluid in continuous contact with the occasionally inflamed everted vaginal mucosa. The ulcers of the uterus are caused by trauma due to manipulation during difficult hygiene maneuvers in obesity and by urethral contamination. To avoid urine contamination, the catheter was applied. The patient is alert, oriented, without strength deficit, BMI 37.44 (Obese class 2 between 35.00 and 39.99), no communicative disability, declivous oedemas, basal hypomobility with some ronco. Enlarged cardiac area, paraphonic tones, batrachian abdomen with poorly reducible umbilical hernia. Malignancy nature of ulcers was excluded through cytological examination of cells taken from the ectocervix and endocervix. The medical history contraindicates surgery.

On medication with Medrol 1 cp, antra 1 cp, lacirex 1 cp, losartan 1 cp, monocinque 20 mg 1 cp × 2, furosemide × 2, allopurinol 300 mg × 2, relvar 1 puff die, tavanor 1 puff die, O2 2 l/m’ at intervals. Unfortunately, drug therapy has not improved the condition of the ulcers of the vaginal ulcers secondary to uterine prolapse. The patient was informed of the possibility of using platelet concentrates for the treatment of uterine ulcers and informed consent was obtained.

## 3 Differential Diagnosis, Investigations and Treatment

In evaluating vaginal ulcers associated with a stage IV uterine prolapse, it is essential to consider an accurate differential diagnosis, as several conditions may present with similar clinical features. First, neoplastic diseases of the lower genital tract must be excluded, particularly cervical and vaginal carcinoma, which may appear as ulcerated or necrotic‐inflammatory lesions; in the case described, malignancy was ruled out through cervical and endocervical cytology. Pressure or friction ulcers should also be considered, as they are common in patients with massive prolapse exposed to air, moisture, and trauma resulting from difficult hygiene, conditions that were exacerbated in our patient by obesity and reduced mobility. Infectious ulcers, such as those caused by herpes simplex virus, syphilis, or chronic bacterial infections, represent additional diagnostic alternatives, although no clinical or laboratory findings suggestive of herpes infection were observed in this case. Overall, the clinical presentation and medical history of our patient, together with the negative cytological examinations, supported the diagnosis of pressure ulcers secondary to long‐standing complete uterine prolapse.

## 4 Conclusion and Results (Outcome and Follow‐Up)

In conclusion, L‐PRF has shown significant potential as an effective treatment for chronic non‐healing vaginal ulcers, offering advantages over conventional dressings in terms of efficacy, safety, and cost‐effectiveness.

**FIGURE 2:**
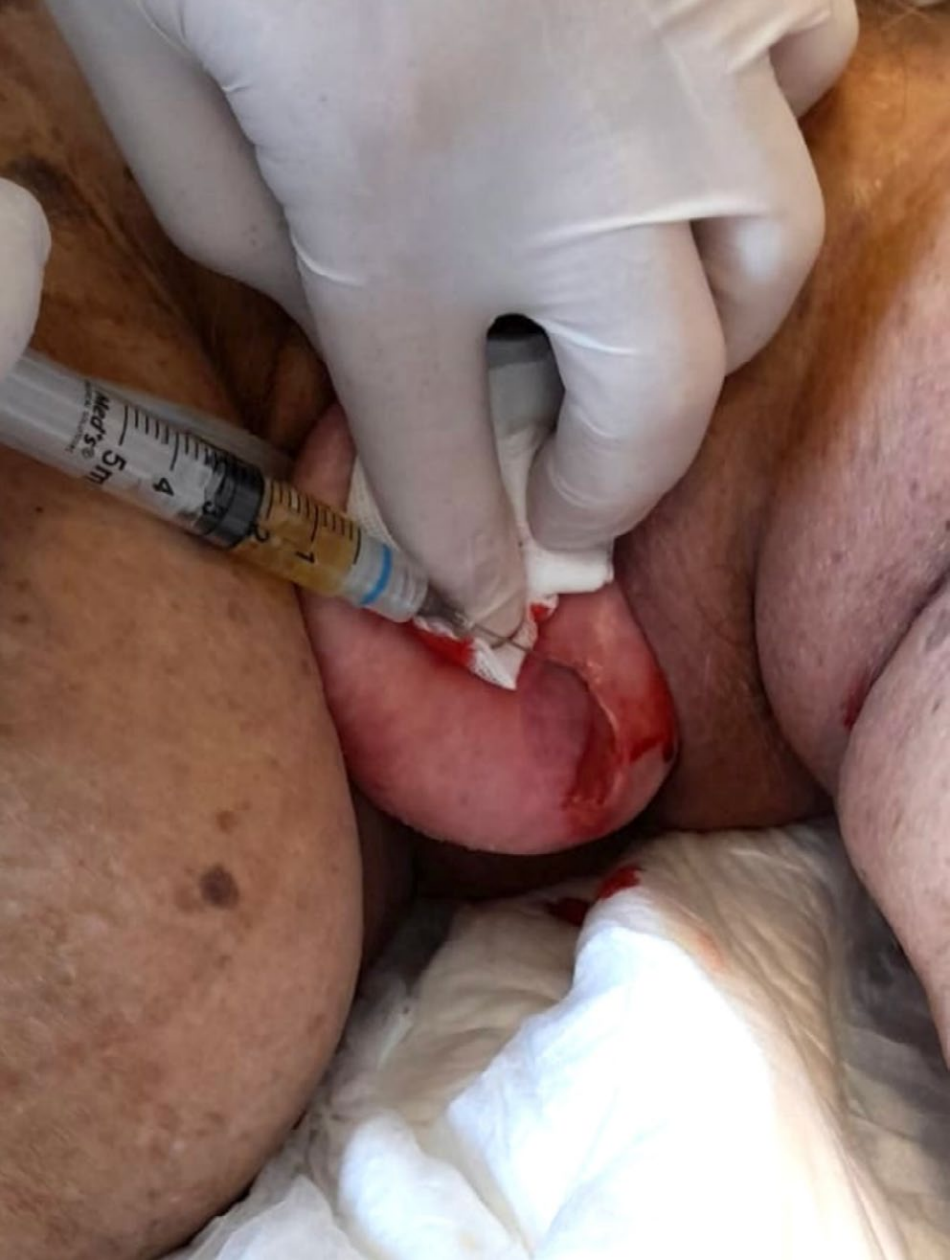
Infiltration of APL in uterine tissue.

**FIGURE 3:**
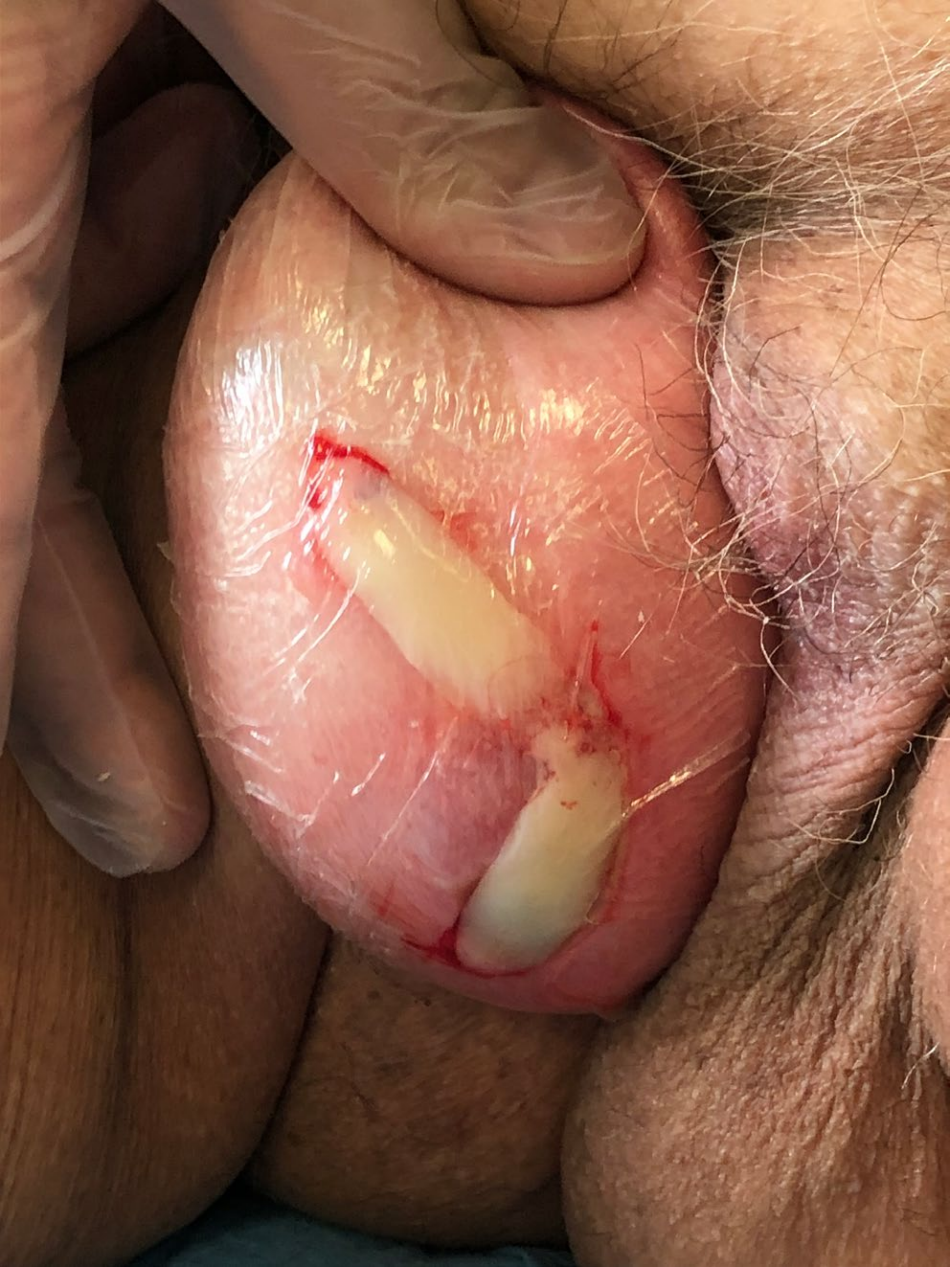
Two APG clots covered the two ulcers.

**FIGURE 4:**
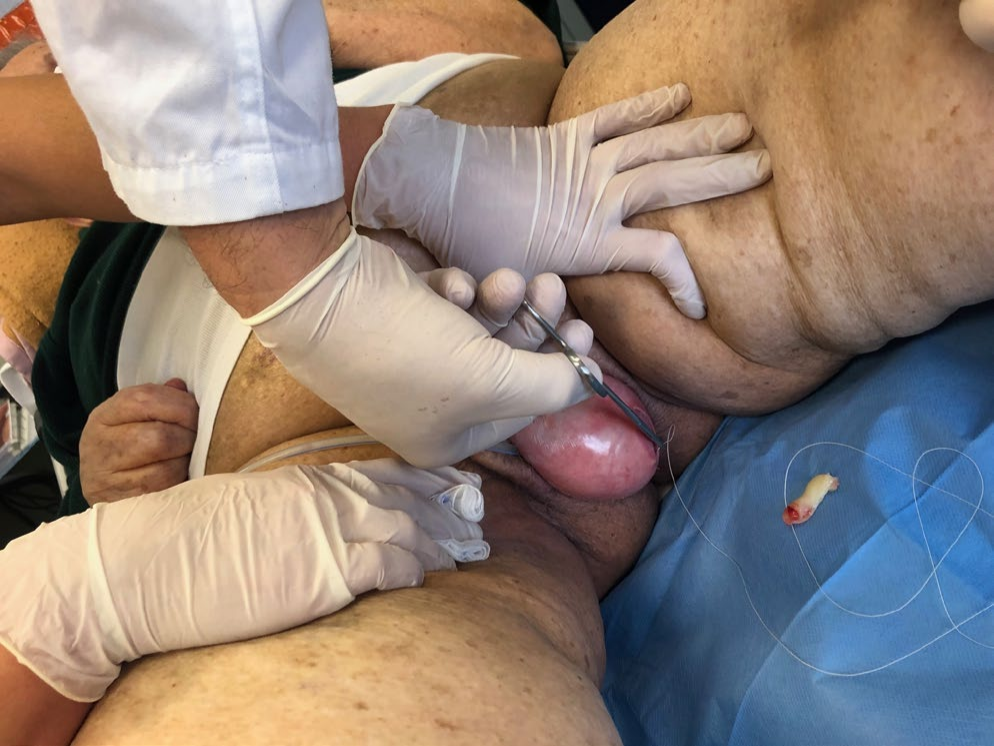
The APG clot was sutured.

The patient was treated in January 2023. The ulcer area was infiltrated with L‐PRF and covered with PRF, which were sutured and protected with an inverted glove (Figures 2, 3, 4).

**FIGURE 5:**
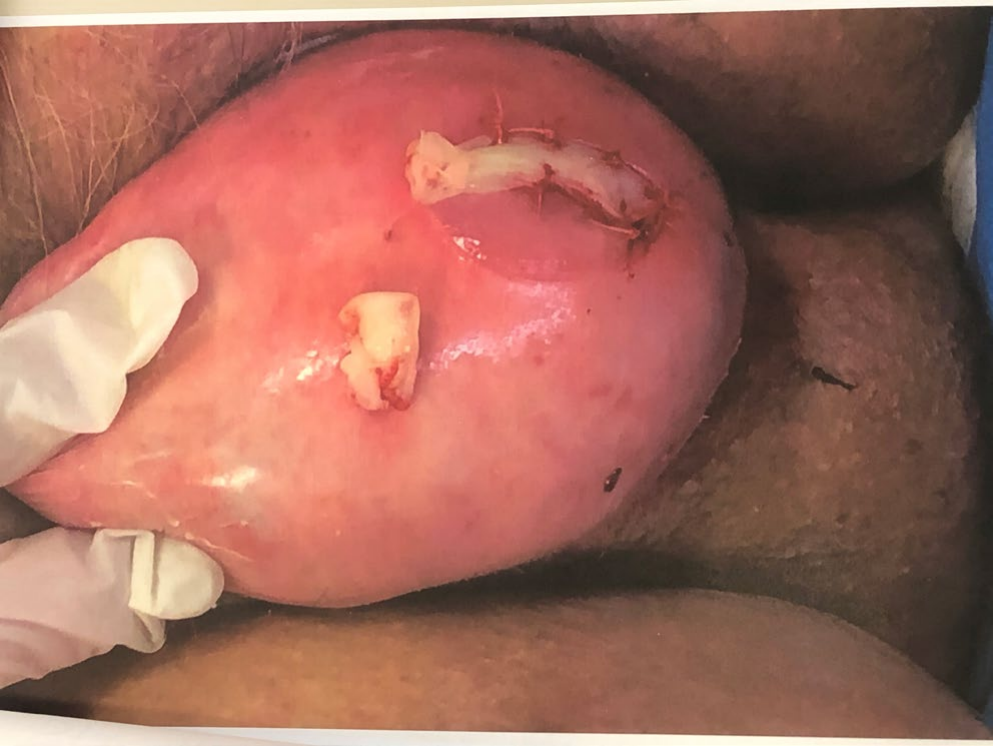
A clear improvement in the ulcers was observed after 2 weeks.

**FIGURE 6:**
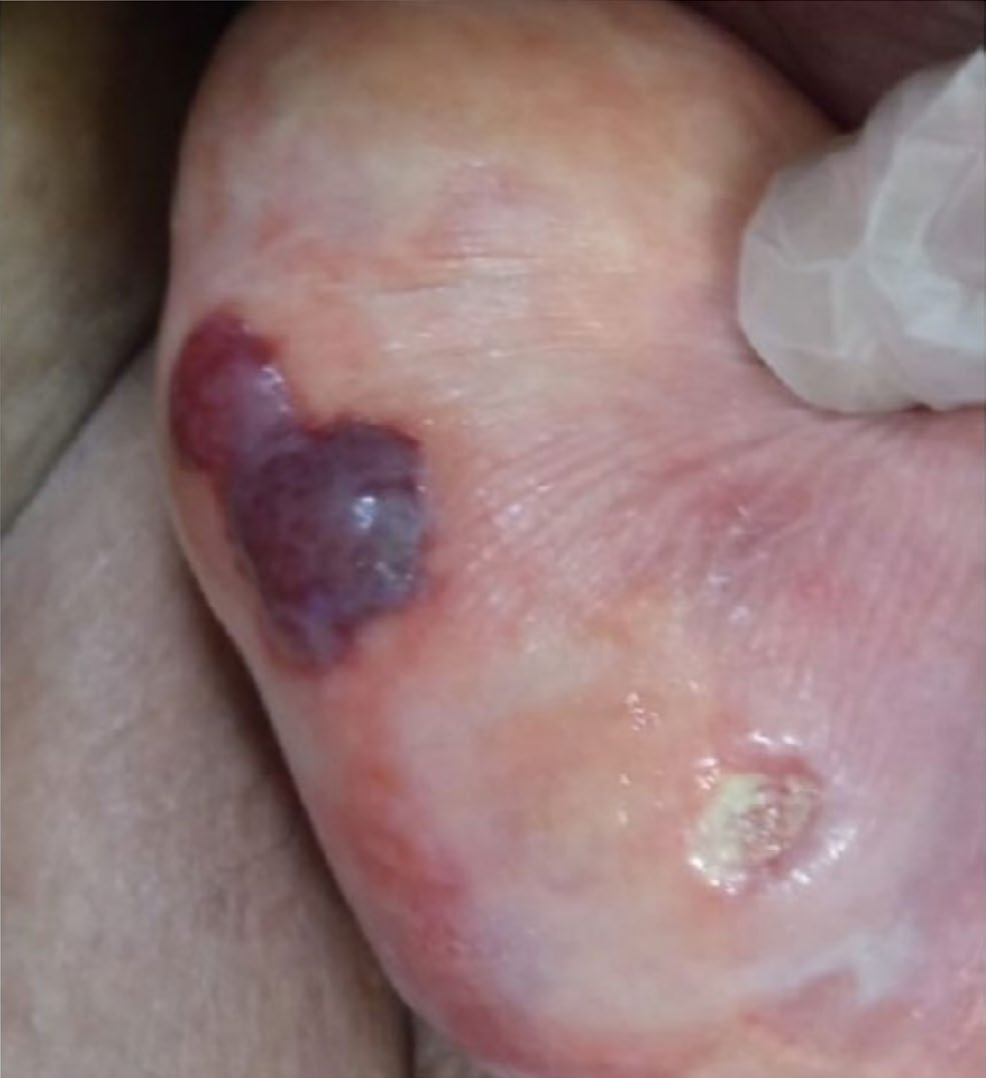
After 3 weeks, complete healing of ulcers was observed.

**FIGURE 7:**
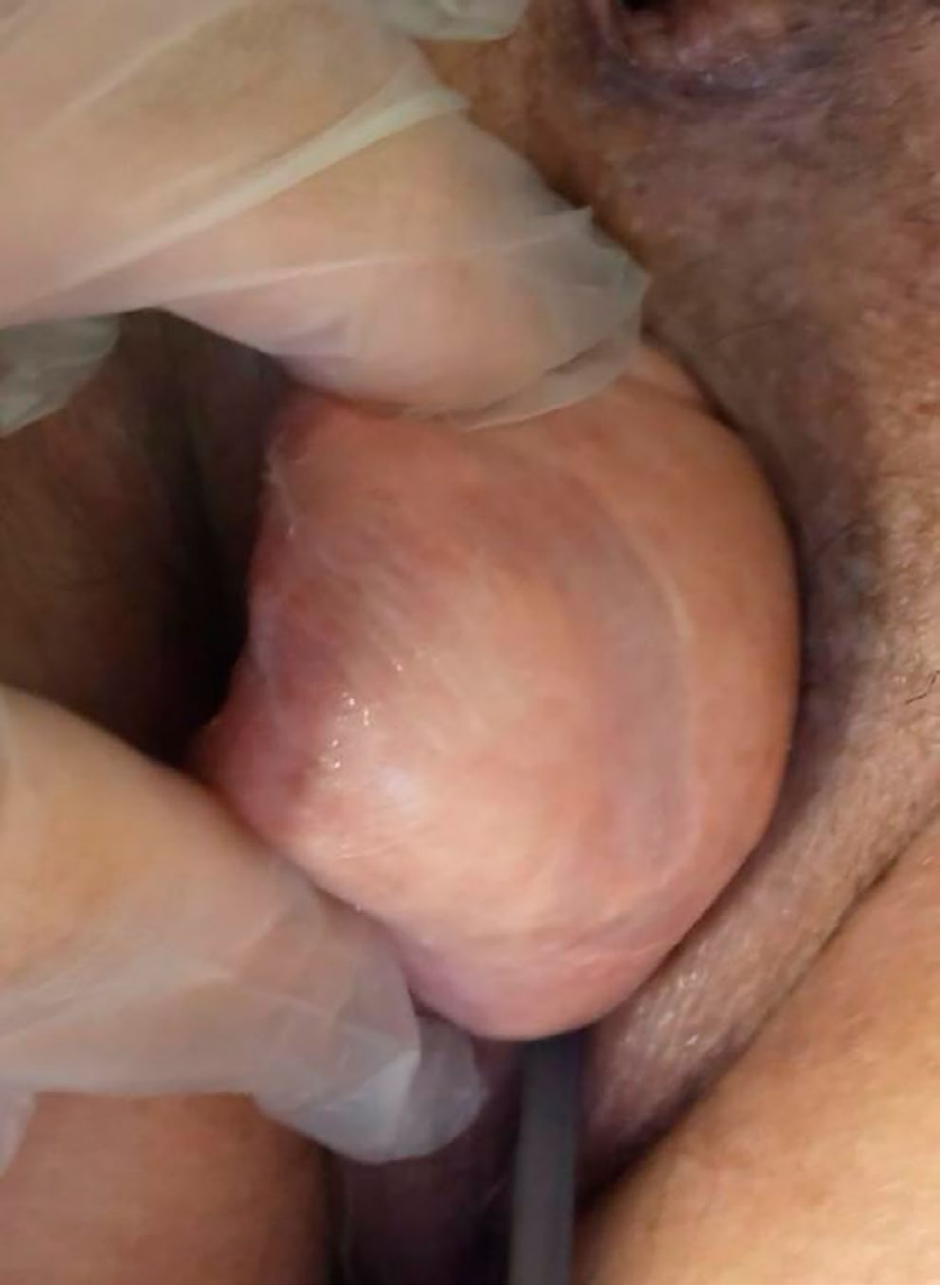
Two new ulcers formed after 2 months of follow‐up.

L‐PRF clot were applied every 15 days until the ulcers had healed and an improvement in the ulcers was observed, one almost completely healed (Figure 5). After two months, complete healing of the two ulcers was observed (Figure 6). A small ulcer recurrence occurred after two months due to decubitus (Figure 6). The patient was followed up for 10 months and no relapses were observed (Figure 7). However, the heart and kidney conditions worsened and the patient died in May 2024. Chronic renal failure was caused by prolonged uterine prolapse.

## 5 Discussion

Pelvic organ prolapse can lead to protrusion through the vaginal opening, resulting from the relaxation and weakening of ligaments, connective tissue, and pelvic floor muscles. This condition is often associated with stress urinary incontinence symptoms, which may negatively affect sexual function and quality of life [27]. The treatment considered the gold standard is colposacropexy (CS), which involves attaching the anterior and posterior vaginal walls to the anterior longitudinal sacral ligament using a mesh. Several surgical approaches are available: open laparotomy or minimally invasive techniques, including laparoscopic and robotic procedures. These methods restore the horizontal axis of the vagina and generally include a concomitant hysterectomy [28, 29, 30].

The results of the present case report show the positive effect of L‐PRF on the uterine ulcers that is a promising treatment for uterine in case of prolapse. Pelvic organ prolapse is often associated with ulcers of the uterus. Leukocyte‐Platelet Rich Fibrin (L‐PRF) is an autologous graft increasingly used in various medical and dental procedures due to its potential to improve healing and tissue regeneration. L‐PRF has emerged as a promising treatment for chronic non‐healing ulcers, demonstrating significant efficacy in promoting wound healing. Several studies have demonstrated the efficacy of L‐PRF in treating chronic non‐healing ulcers. For instance, a study involving 50 patients with chronic non‐healing ulcers showed significant improvement in wound healing with weekly L‐PRF dressings over four weeks [31]. The mechanism of action of platelet concentrate on tissue repair has been investigated through in vivo and in vitro study. L‐PRF is an autologous biomaterial composed of a fibrin matrix polymerized in a tetramolecular structure, incorporating platelets, leukocytes, cytokines, and circulating stem cells. This three‐dimensional structure facilitates the migration of endothelial cells and fibroblasts, promoting rapid angiogenesis and remodeling into a more resistant connective matrix. Platelet concentrate (PC) has a high concentration of growth factors, such as PDGF, TGF‐β, EGF, FGF, and VEGF, that play a critical role in tissue healing and regeneration, including bone, tendons, cartilage, muscles, and ligaments [32]. PC treatment has also been used in the treatment of vulvar lichen sclerosus, lichen planopilaris, hair loss, soft tissue augmentation, and other medical conditions. PC also plays a positive role in the rejuvenation of tissue and wound healing. PC has been used for intrauterine adhesions (IUAs) treatment intrauterine infusion of PRP, intrauterine [33]. PRP is a non‐transfusion haemocomponent that has platelet counts that are four to six times upper than those of whole blood. Also, L‐PRF has been shown to increase endometrial healing/regeneration and pregnancy outcomes in vitro and in animal study [34]. Animal experiments show that autologous PRP stimulated and accelerated the regeneration of the endometrium and also decreased fibrosis in a murine model of damaged endometrium. L‐PRF has been used in various gynecological conditions, including thin endometrium, intrauterine adhesions, and recurrent implantation failure [35]. Clinical studies have demonstrated that L‐PRF can improve endometrial function, increase fertility, and enhance tissue regeneration [34]. In fact, a study on rats showed that L‐PRF application maintained promoted endometrial regeneration, uterine structure, and decreased fibrotic areas [34].

Uterine prolapse is a condition with varying degrees of severity, classified into four stages. The prevalence and risk factors are well‐documented, with multiparity and advanced age being significant contributors. Symptoms range from mild discomfort to severe complications, necessitating a range of treatment options from conservative management to surgical interventions. The impact on quality of life is profound, highlighting the importance of effective diagnosis and treatment to alleviate symptoms and improve patient outcomes. Pelvic examination is fundamental for the diagnosis and staging of UP.

Vaginal hysterectomy (VH) has long been considered the gold standard approach for the surgical management of UP. However, VH alone does not ensure the restoration of the cardinal‐uterosacral ligament complex, and several adjunctive procedures have been proposed to avoid recurrences.

The McCall culdoplasty was introduced in 1957 as a technique to correct enterocoele [36].

The technique included the suspension of the vault into the origins of the uterosacral ligaments with the obliteration of the cul‐de‐sac peritoneum. Even if ureteric injury was reported as being an important complication of the technique, it can be avoided by means of some preventive procedures [37]. SSF (sacrospinous ligament fixation), suturing the cardinal and deep uterosacral ligaments to the vaginal cuff, or high circumferential obliteration of the Douglas' pouch are other recommended adjunctive techniques for uterovaginal prolapse management [38].

Uterine preservation surgery can be considered in women who wish to maintain fertility or desire to retain their uterus [39].

An alternative approach to VH was described by Archibald Donald since 1888.

The original Manchester repair surgery consisted of a cervical amputation with mobilization and attachment of the cardinal ligaments anterior to the cervix, followed by an anterior colporrhaphy to support the vagina. Subsequent cervical incompetence may lead from preterm deliveries and cervical stenosis to mechanical dysmenorrhea and secondary infertility. Due to these complications, nowadays this technique is largely obsolete [38].

Other techniques for uterine preservation were developed using a vaginal, abdominal, or laparoscopic approach. Sacrospinous ligament fixation, uterosacral ligament suspension, or sacrohysteropexy/sacrocolpopexy are other main techniques used for the management of UP.

Abnormal bleeding or precancerous cervical lesions represent important contraindications to uterine preservation and patient must be aware that a future pregnancy can jeopardize the results achieved.

Obliterative surgery for the treatment of vaginal vault prolapse can be associated with the maintaining of the uterus (LeFort colpocleisis) or with a previous or concomitant hysterectomy. They are typically reserved for advanced stages of prolapse in older patients no longer wishing to be sexually active [40]. Low rates of perioperative morbidity, short operative time, and low risk of recurrence are the main advantages of obliterative treatments [38].

This is especially true when considering the inclusion of a woman with medical risk factors: previous prolapse surgery [2], obesity (BMI > 29), chronic bronchopneumonia, chronic straining during defecation, acute heart failure, suffering from umbilical hernia, hypertension, hyperuricemia, polyhydric arthrosis. Our study also showed reduction of pain, urinary and bowel symptoms over time. However, there is not confirmation supporting the use of Leukocyte‐Platelet Rich Fibrin when used as graft for treating uterine prolapse. However, LPRF when used as an adjunct therapy during vaginal prolapse surgery promotes tissue regeneration and healing, thus improving surgical outcomes [41]. A recent research shows that Platelet Rich Plasma (PRP) offers direct evidence for treating uterine scar defects, and promising new treatment options to address female infertility, but not regarding the treatment of uterine prolapse [42]. L‐PRF is a natural autologous dressing with no side effects, inexpensive (costing 1.5€ per tube) which, as reported elsewhere in vulnogamic areas, acts as a healing booster in non‐healing wounds despite advanced dressings. Despite the limitations of the present study, this case report is noteworthy and offers a new perspective for the treatment of inoperable patients affected by uterine prolapse associated with vaginal ulcers. It would be interesting to confirm these findings in additional case reports. In conclusion, L‐PRF has shown significant potential as an effective treatment for chronic non‐healing vaginal ulcers, offering advantages over conventional dressings in terms of efficacy, safety, and cost‐effectiveness.

## Author Contributions

**Alberta Greco Lucchina:** supervision, validation, visualization. **Enrico Rescigno:** software, supervision, visualization. **Canata Alessandra:** software, supervision, validation. **Gianluca Nicolai:** visualization, writing – original draft, writing – review and editing. **Antonio Scarano:** validation, writing – original draft.

## Funding

The authors have nothing to report.

## Ethics Statement

Written consent has been obtained. The Lavagna Hospital (Ge) ASL 4 Liguria, Italy, classified the study to be exempt from ethical review as it is not carrying risk and involves the use of existing data that contains only non‐identifiable data about human beings. A consent to participate was requested for this case report. The patient was fully capable of understanding the consent process at the time of signing.

A family member was present during the discussion and consent procedure.

Written informed consent was obtained for both the treatment and the publication of clinical images.

## Consent

Written consent was obtained for the publication of photos and data.

## Conflicts of Interest

The authors declare no conflicts of interest.

## Data Availability

The data supporting this study's findings are available in the manuscript.
